# Graft patency at 3 months after off- and on-pump coronary bypass surgery: a randomized trial

**DOI:** 10.1007/s12055-019-00869-0

**Published:** 2019-10-28

**Authors:** Lokeswara Rao Sajja, Kunal Sarkar, Gopichand Mannam, Venkata Krishna Kumar Kodali, Chandrasekar Padmanabhan, Sanjeeth Peter, Anvay Mulay, Prashanthi Beri

**Affiliations:** 1 Division of Cardiothoracic Surgery, STAR Hospitals, Road no. 10, Banjara Hills, Hyderabad, Telangana 500 034 India; 2 Division of Cardiovascular Surgical Research, Sajja Heart Foundation, Srinagar Colony, Hyderabad, 500073 India; 3 Division of Cardiothoracic Surgery, Medica Superspeciality Hospital, 127-Mukundapur, EM bypass, Kolkata, 700025 India; 4 Division of Cardiothoracic Surgery, Krishna Institute of Medical Sciences, 1-8-31/1,Minister Road, Secunderabad, Telangana 500003 India; 5 Division of Cardiothoracic Surgery, G Kuppuswamy Naidu Memorial Hospital, Pappanaicken Palayam, Coimbatore, 641 037 India; 6 Division of Cardiothoracic Surgery, DDMM Heart Institute, Mission Road, Nadiad, Gujarat 387002 India; 7 Division of Cardiothoracic Surgery, Fortis Hospital, Multi-Specialty Hospital Mulund West, Mumbai, 400078 India; 8 Division of Clinical Research, Sajja Heart Foundation, Srinagar Colony, Hyderabad, 500073 India

**Keywords:** Coronary artery bypass grafting (CABG), Multidetector computerized tomographic angiography, Coronary angiography

## Abstract

**Purpose:**

Coronary artery bypass grafting (CABG) is performed either with the aid of cardiopulmonary bypass (on-pump) or without cardiopulmonary bypass (off-pump). There is a scarcity of angiographic data to support the non-inferiority of off-pump technique to on-pump technique. The objective of this study is to ascertain the non-inferiority of off-pump CABG when compared to on-pump CABG in terms of angiographically assessed graft patency at 3 months.

**Methods:**

A total of 320 patients with multivessel coronary artery disease were enrolled in a multicenter prospective randomized trial either to on-pump CABG (*n* = 162) or off-pump CABG (*n* = 158) between March 2016 through March 2017. Graft patency was evaluated by using either multidetector computerized tomographic angiography or conventional coronary angiography at 3 months. The major adverse cardiac and cardiovascular events (MACCE) were also analyzed at 3 months.

**Results:**

The median number of grafts per patient in off-pump was 3.00 (Q1:3.00 and Q3:4.00) vs on-pump 4.00 (Q1:3.00 to Q3:4.00), and the mean number of grafts per patient was lower in the off-pump CABG at 3.45 ± 0.75 vs 3.64 ± 0.70 in the on-pump CABG (*p* = 0.01). There was no significant difference in mortality at 3 months between the off-pump (0.63%) and on-pump groups (1.85%) with *p* value of 0.62. The cumulative combined MACCE showed significant difference between off-pump group (0.63%) and on-pump group (5.55%), *p* = 0.01. Follow-up angiograms were done in 239 (75%) patients with 120 off-pump and 119 in the on-pump group. The analysis was also done regarding graft patency in a graded manner—when analysis of A (excellent) grafts vs B (stenosed) grafts and O (occluded) grafts were made, there was no statistically significant difference in overall graft patency at 3 months between on-pump [376 /429 grafts (87.6%)] and off-pump [366 /420 grafts (87.1%)] groups (*p* = 0.82). The patency rates were similar among bypass conduits (left internal thoracic artery (ITA) in off-pump (91.4%) vs on-pump (92.9%) *p* = 0.66, right ITA in off-pump (82.1%) vs on-pump (81.8%) *p* = 0.97, radial artery in off-pump (84.4%) vs on-pump (82.6%) *p* = 0.81; saphenous vein in off-pump (85.8%) vs on-pump (86.3%), *p* = 0.86 and among 3 coronary territories.

**Conclusions:**

Off-pump CABG is non-inferior to on-pump CABG in terms of overall graft patency at 3 months and was associated with a fewer combined cumulative MACCE compared to on-pump CABG.

**Electronic supplementary material:**

The online version of this article (10.1007/s12055-019-00869-0) contains supplementary material, which is available to authorized users.

## Introduction

CABG is the preferred method of treatment for patients with multivessel coronary artery disease (CAD) and is performed either with the aid of cardiopulmonary bypass (on-pump) or without cardiopulmonary bypass (off-pump) [1]. Since the beginning of off-pump CABG, there are concerns regarding the quality of coronary anastomosis and the completeness of revascularization, particularly of the lateral wall of the left ventricle. Currently, about 10–20% of CABG procedures are being performed using off-pump technique in North America and Europe [2]. The data on lower graft patency in patients undergoing off-pump CABG emanates from centers with low adoption of off-pump technique [1, 3–5], but graft patency was reported to be similar with either off-pump or on-pump technique by the surgeons whose adoption rate of off-pump technique was over 50% of the cases [6].

Off-pump CABG is adopted in over 50% of the patients in India [7], whereas it is on decline in the Western world. In India, several surgeons adopt an on-pump strategy only when the patient is ineligible for off-pump because of hemodynamic instability or diffuse CAD with small caliber vessels. This study was contemplated to assess whether the quality of revascularization in terms of graft patency in off-pump is non-inferior to that of on-pump CABG by angiographic assessment when the procedure is performed by the surgeons with off-pump adoption rates in excess of 50% and have performed over 250 off-pump CABG procedures and negotiated the learning curve prior to participation in the trial.

## Patients and methods

This Prospective Randomized comparison of Off-pump and On-pump Multivessel coronary artery bypass surgery To Evaluate outcomes and graft patency (PROMOTE patency) trial was conducted between March 2016 through March 2017. The patients who met the inclusion and exclusion criteria were randomized in the ratio of 1:1 using block randomization, with a block size as 4, using software SAS version 9.2. A total of 321 patients were randomized and 320 patients (recruitment ranged from 26 to 56 patients per surgeon) were enrolled to either on-pump (*n* = 162) or off-pump (*n* = 158) CABG at 6 centers by 7 surgeons in India.

The PROMOTE patency trial was registered in the Clinical Trials Registry of India (CTRI/2017/10/010030). This trial complies with the principles of The Declaration of Helsinki and was approved by the institutional ethics committees by the participating institutions and all patients gave an informed written consent to participate in the study.

The inclusion criteria were male or female aged ≥ 21 years and ≤ 70 years, and multivessel CAD, with triple vessel disease or left main coronary artery (LMCA) stenosis, requiring isolated CABG and with left ventricular ejection fraction (LVEF) of ≥ 40%. Exclusion criteria were CABG with concomitant procedures, contra-indications to either off-pump or on-pump CABG, chronic atrial fibrillation, and serum creatinine > 1.3% mg/dL. Graft patency was assessed at 3 months by either 128 slice multidetector computed tomographic angiography (MDCT) or conventional catheter coronary angiography (CAG). Graft patency was evaluated on the basis of type of conduit and coronary artery territory, as well as the number of anastomoses patent per patient. The graft evaluation was made similar to Fitzgibbon grading of grafts [8]: A (excellent) vs B (stenosed) and O (occluded). In this study, graft patency was graded as A (excellent patency) vs B (partially stenosed) and O (occluded) grafts. All the graft angiograms (MDCT and CAG) were analyzed at the trial coordinating center by a single team comprising of a cardiologist and a radiologist with knowledge of the number and distribution of grafts performed but blinded for the type of revascularization technique used(on-pump or off-pump). The occurrence of MACCE was recorded as secondary outcomes at first and third month postoperatively.

Surgeons who had performed more than 500 CABG procedures (250 procedures of each technique) during the 3 years prior to the trial with 50% adoption of the off-pump technique and who had a post CABG mortality rate ≤ 3% for isolated primary CABG were selected for this study.

### Statistical methods

In order to detect a difference of 10% in patency rates between off-pump and on-pump CABG, with a power of 90% and one tailed, 5% significance level, and assuming patency rates of 80% and 90%, a total of 1050 distal graft anastomoses (525 grafts per group) were required. Since each patient had at least three grafts, a total of 310 patients would be necessary (155 patients in each arm.) To compensate for eventual dropouts of 10%, the study kept an enrolment target of 350 patients (175 patients in each arm).

ROOBY trial [1] reported lower graft patency with off-pump compared to on-pump CABG (82.6 vs 87.8%, respectively; *p*_0.001) at 1 year. Khan et al. [3] reported the overall patency rate for grafts performed with on-pump was significantly higher than the patency rate for those performed off-pump (98 vs 88%, *p* = 0.002) [absolute difference of 10% with 95% CI (3.8 to 16.2)] at 3 months. The sample size in this study was calculated based on the graft patency of the above trials. The current study is a non-inferiority trial, and hence, the null hypothesis is formulated as H0: The graft patency rate for off-pump is inferior to on-pump graft patency rate by a clinically relevant patency rate of 10%. HA: The graft patency rate for off-pump is non-inferior to the graft patency rate of on-pump or, alternatively is formulated as H0: The difference in the graft patency rate between off-pump and on-pump is (− 10%) HA: The difference in the graft patency rate between off-pump and on-pump is (> − 10%). The required sample size (*n*) for each group has been arrived assuming an α = 5% or a confidence level (1-a) of 95%, ß = 10% or a power (1-ß) of 90%, the non-inferiority clinical margin (d) of 10%, and Zx is the standard normal variate for a one sided x.

Statistical analysis was performed by the trial coordinating center using software SAS version 9.2. Continuous variables have been expressed as median, mean ± standard deviation. The categorical variables have been expressed as raw numbers and percentages. The differences were analyzed with chi-square, Fisher exact test (if cell frequency was less than 5), and two sample *t* test. The statistical significance for all the tests was accepted at a probability level < 0.05.

### Preoperative optimization of medical therapy

Withdrawal of antiplatelet drugs was done 4 days prior to surgery and low molecular weight heparin was started.

### Surgical techniques

Premedication, anesthetic protocols, surgical access to the heart via a standard median sternotomy, conduit harvesting techniques, and distal and proximal anastomotic techniques were similar between the groups. Heparin dose was adjusted to achieve target activated clotting time of > 480 s and > 350 s for on-pump and off-pump groups respectively. Heparin was reversed using protamine at a dose of 1.3 mg/100 IU heparin in both the groups. The surgical techniques of revascularization were followed as described earlier [9].

#### On-pump technique

Myocardial protection was achieved by cold (4 °C) antegrade blood and potassium cardioplegia. The distal coronary anastomoses were performed with either 7–0 or 8–0 polypropylene continuous suturing techniques and the proximal anastomoses were performed using 6–0 polypropylene sutures as described earlier [9].

#### Off-pump technique

The method of exposure and stabilization of heart to perform distal coronary anastomoses consisted of the technique previously described [9]. Target artery stabilization was achieved with vacuum stabilizers - Octopus 4 or Evolution (Medtronic Inc., Minneapolis, MN, USA) or ACROBAT-i Stabilizer System (Maquet GmbH & Co, Rastatt, Germany), and clearview intra coronary shunts were used [Medtronic Inc. Minneapolis, MN, USA] in all coronary arteries measuring more than 1.25 mm in diameter while constructing the distal anastomoses. Visualization of the anastomotic area was enhanced by using humidified carbon dioxide blower/mister (Medtronic Inc. Grand Rapids, Mich) to disperse the blood from the site of distal anastomosis. Postoperatively, all patients received dual antiplatelet therapy (aspirin 75 mg, clopidogrel 75 mg) once daily. Patients in whom endarterectomy was performed on any of the coronary arteries received acenocoumarol with target international normalized ratio (INR) of around 2.0 for 3 months.

### Outcome measures

The primary endpoint was graft patency at 3 months, assessed by either 128 slice MDCT or CAG. Graft assessment was done similar to Fitzgibbon grading of ABO grade grafts [8]. Grade A graft was defined as an excellent graft with unimpaired runoff, and grade B graft was defined as stenosis reducing caliber of proximal or distal anastomoses or trunk to 50% of the grafted coronary artery. Grade O graft was defined as absence of contrast material along the course of the graft, through the graft anastomosis to the native coronary artery or to the following graft segment and native vessel. In sequential bypass grafts, each anastomosis of the graft was counted as an individual graft. The secondary end points were death, nonfatal cerebrovascular stroke/accident (CVS/CVA), myocardial infarction (MI), and repeat revascularisation at 30 days and 3 months postoperatively. The occurrence of MACCE was defined as death, MI, or CVS at 30 days and 3 months after CABG. The terminologies used in the study have been defined. Cardiovascular (CV) death: All deaths were considered cardiovascular unless a specific noncardiovascular cause is evident (e.g., malignancy). CVS: new acute focal neurological deficit (except for subarachnoid hemorrhage which may not be focal) thought to be of vascular origin with signs or symptoms lasting greater than 24 h. MI perioperative (within 24 h of surgery): new pathologic Q waves with documented new wall motion abnormalities other than septal wall or cardiac markers = 10xULN (upper limit of normal). MI nonperioperative (later than 24 h of after surgery): electrocardiogram (ECG) changes consistent with infarction (new significant Q waves in two contiguous leads in the absence of previous left ventricular hypertrophy (LVH) or conduction abnormalities) or evolving ST segment to T – wave changes in two contiguous leads or new left bundle branch block or ST segment elevation requiring thrombolysis or percutaneous coronary intervention (PCI) and cardiac markers (troponins or creatinine kinase muscle/brain (CKMB)) in the necrosis range. Repeat coronary revascularization: new CABG procedure or PCI associated with documented ischemia by stress testing (ECG, echo, or nuclear) and graft failure or new culprit lesion) = 70% luminal stenosis). Deep sternal wound infections: bone-related or any drainage of purulent material from the sternotomy wound and instability of the sternum.

## Results

**Fig. 1:**
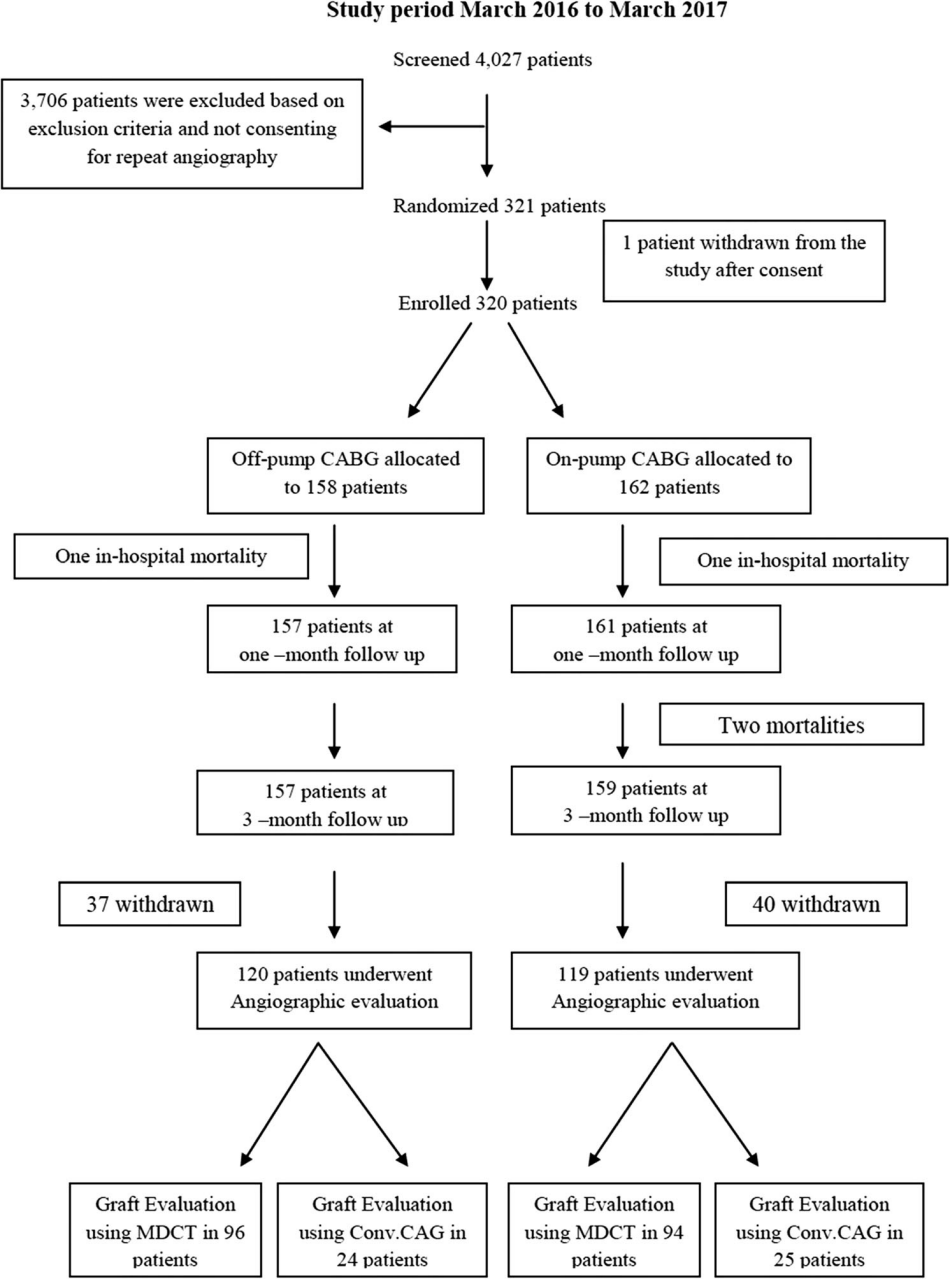
Study flow chart

A total of 320 patients were enrolled and were randomly assigned to either off-pump CABG (*n* = 158 patients) or on-pump CABG (*n* = 162 patients). Of 320 patients, 318 patients at 1 month and 316 patients at 3 months survived and were analyzed for MACCE. Among the survivors, 239 (75.6%) patients (120 in off-pump group and 119 in on-pump group) underwent graft angiographic evaluation (MDCT in 190 patients and CAG in 49 patients at 3 months (Fig. 1) and angiographic evaluation was declined by 77 survivors.

Baseline variables, intraoperative (Table 1) and postoperative variables (Table 2) were comparable between the groups. The incidence of asymptomatic carotid artery stenosis was 0.63% (1 patient) in off-pump and 0.62% (1 patient) in on-pump group, *p* = 1.00 in our study (Table 1). All patients in the trial had EF ≥ 40%. Euroscore II was calculated for all patients and the median score is less than 1 (off-pump group 0.98 and on-pump group 0.98) which indicates a low-risk population in both groups. Two patients (1.27%) were converted from off-pump to on-pump CABG and none was converted from on-pump to off-pump technique. The results were analyzed as intention to treat analysis. A total of 1137 grafts were used in 320 patients. The median number of grafts per patient in off-pump was 3.00 (Q1:3.00 and Q3:4.00) vs on-pump 4.00 (Q1:3.00 to Q3:4.00), and mean number of grafts/patient was off-pump 3.45 ± 0.75 vs on-pump 3.64 ± 0.70) (*p* = 0.01) (Table 1). One hundred forty-nine (94.30%) patients received left internal thoracic artery (LITA) graft in off-pump and 153 (94.44%) in on-pump CABG group, *p* = 0.95; 22 (13.92%) patients received right ITA graft in the off-pump and 17 (10.49%) in on-pump group, *p* = 0.34. A total of 90 (28%) patients received two or more arterial grafts (right internal thoracic artery (RITA)/radial artery). Right ITA in 39 patients and as in situ graft in 15 patients and as a free graft in 24 patients. There was no significant difference in the number of sequential anastomoses between the two groups (25.95% grafts in off-pump and 23.78% in on-pump group; *p* = 0.46). The distribution of grafts among various territories is mentioned in (Table 3). No difference was observed between the groups in terms of index of completeness of revascularization (number of grafts performed divided by number of grafts intended) 0.98 vs 1.00 (*p* = 0.8).

**Table 1:** Baseline and intra-operative variables compared between off-pump and on-pump CABG

Variable		Off-pump CABG (*n* = 158)		On-pump CABG (*n* = 162)		*p* value
		Number	%	Number	%	
Age (mean ± SD)		58.01 ± 7.06		58.8 ± 7.2		0.31
Sex	Females	19	12.03	17	10.49	0.66
	Males	139	87.97	145	89.51	
Diabetes		87	55.06	100	61.73	0.22
Hypertension		108	68.35	102	62.96	0.31
Smoking		23	14.56	20	12.35	0.56
Dyslipidemia		93	58.86	90	55.56	0.55
Carotid artery stenosis > 60%*		1	0.63	1	0.62	1.00
Transient ischemic attacks		2	1.27	0	0.00	–
COPD*		4	2.53	2	1.23	0.44
Myocardial infarction		51	32.28	49	30.25	0.69
Left main disease		22	13.92	28	17.28	0.40
Prior PTCA		8	5.06	7	4.32	0.75
Blood usage		77	48.73	89	54.94	0.266
Number of grafts	(mean ± SD)	3.45 ± 0.75		3.64 ± 0.70		0.01
	Median	3.00 (Q1:3.00 and Q3:4.00)		4.00 (Q1:3.00 to Q3:4.00)		

In off-pump CABG, conversion was done in 2 patients (1.27%)

In on-pump CABG, means of CPB time and aortic cross clamp time were 87.54 ± 30.76 and 52.93 ± 17.71 min respectively

*Fisher’s exact test was used

*CABG*, coronary artery bypass grafting; *COPD*, chronic obstructive pulmonary disorder; *CPB*, cardiopulmonary bypass; *IABP*, intra-aortic balloon pump; *NYHA*, New York Heart Association; *PTCA*, percutaneous transluminal coronary angioplasty

**Table 2:** In-hospital outcomes compared between off-pump and on-pump CABG

Variable	Off-pump CABG (*n* = 158)		On-pump CABG (*n* = 162)		*p* value
	Number	%	Number	%	
Re-exploration*	4	2.53	5	3.09	> 0.99
Intra and post-operative IABP*	2	1.27	6	3.70	0.28
Post-operative CVA	0	0.00	0	0.00	–
AKI requiring RRT	0	0.00	0	0.00	–
Pulmonary complications	0	0.00	3	1.85	–
GI complications	0	0.00	1	0.62	–
Post-operative atrial fibrillation	15	9.49	9	5.56	0.18
DSWI*	1	0.63	2	1.23	> 0.99
Post-operative MI	0	0.00	0	0.00	–
Re-revascularization	0	0.00	0	0.00	–
ICU stay (mean ± SD)	2.75 ± 2.12		2.74 ± 1.83		0.87
Ward stay (mean ± SD)	4.02 ± 1.09		4.14 ± 1.44		0.47
Death*	1	0.63	1	0.62	> 0.99

*Fisher’s exact test was used

*AKI RRT*, acute kidney injury requiring renal replacement therapy; *DSWI*, deep sternal wound infection; *GI*, gastrointestinal; *IABP*, intra-aortic balloon pump; *ICU*, intensive care unit; *MI*, myocardial infarction

**Table 3:** Distribution of grafts to various coronary artery territories

Graft distribution (1137 grafts) in the entire cohort of patients (320)				Graft distribution (849 grafts) in the patients (239) underwent graft evaluation			
Conduits	Territories*	Off-pump CABG	On-pump CABG	Conduits	Territories*	Off-pump CABG	On-pump CABG
LITA (347)	LAD (322)	152	170	LITA (256)	LAD (236)	116	120
	LCx (19)	15	4		LCx (16)	12	4
	RCA (6)	3	3		RCA (4)	1	3
RITA (73)	LAD (15)	10	5	RITA (50)	LAD (11)	7	4
	LCx (41)	19	22		LCx (26)	13	13
	RCA (17)	9	8		RCA (13)	8	5
RA (115)	LAD (7)	5	2	RA (91)	LAD (6)	5	1
	LCx (59)	29	30		LCx (48)	22	26
	RCA (49)	24	25		RCA (37)	18	19
SVG (602)	LAD (109)	48	61	SVG (452)	LAD (79)	37	42
	LCx (261)	120	141		LCx (200)	94	106
	RCA (232)	113	119		RCA (173)	87	86
Total conduits 1137	Total territories* 1137	547	590	Total conduits 849	Total territories 849	420	429

*Territories: LAD (LAD+D1 + D2 + D3)

LCx (ramus intermedius, OM1, OM2, OM3, LPDA, distal circumflex)

*RCA*, (distal RCA, Mid RCA, PDA, PLVB); *CABG*, coronary artery bypass grafting; *D*, diagonal; *LAD*, left anterior descending artery; *LCx*, left circumflex artery; *LITA*, left internal thoracic artery; *LPDA*, left posterior descending artery; *PDA*, posterior descending artery; *PLVB*, postero lateral ventricular branch; *RA*, radial artery; *RCA*, right coronary artery; *RITA*, right internal thoracic artery; *SVG*, saphenous vein graft

A total of four deaths occurred in the study. One mortality occurred in off-pump group (postoperative period) and 3 in on-pump group (1 postoperative period and 2 at 3 months). Three deaths were due to noncardiovascular cause and one due to pneumonia. There is no significant difference in mortality at 3 months between off-pump (0.63%) and on-pump groups (1.85%) odds ratio: (0.33, CI 0.03–3.28, *p* = 0.62). There was no significant difference between the groups in the outcome of nonfatal MI [none in off-pump and 1.85% (3 patients) in on-pump group (*p* = 0.24), CVS [0.00% in off-pump and 1.85% (3 patients) in on-pump group (*p* = 0.24). Cumulative combined MACCE in off-pump group (0.63%) was significantly lower than on-pump group (5.55%) with an odds ratio of 0.108 (CI 0.01–0.86, *p* = 0.01). At 3 months follow-up, off-pump CABG was associated with a fewer MACCE compared to on-pump CABG (Table 4).

**Table 4:** Cumulative MACCE between off-pump and on-pump CABG at 3 months

Variables	Technique of CABG (no of patients)	Yes *n* (%)	No *n* (%)	Rate	Risk ratio (95% CI)	Odds	Odds ratio (95% CI)	*p* value
Mortality	Off-pump CABG (158)	1 (0.63)	157 (99.36)	0.0063	0.34 (0.03–3.25)	0.064	0.337 (0.03–3.28)	0.62
	On-pump CABG (162)	3 (1.85)	159 (98.14)	0.0185		0.0018		
Non-fatal MI	Off-pump CABG (158)	0 (0.00)	158 (100.00)	0	0	0	0	0.24
	On-pump CABG (162)	3 (1.85)	159 (98.14)	0.018		0.0189		
CVA	Off-pump CABG (158)	0 (0.00)	158 (100.00)	0	0	0	0	0.24
	On-pump CABG (162)	3 (1.85)	159 (98.14)	0.018		0.0189		
Total MACCE	Off-pump CABG (158)	1 (0.63)	157 (99.36)	0.006	0.11 (0.01–0.88)	0.064	0.108 (0.01–0.86)	0.01
	On-pump CABG (162)	9 (5.55)	153 (0.94)	0.055		0.058		

*CABG,* coronary artery bypass grafting; *CVA*, cerebrovascular accident; *MACCE*, major adverse cardiac and cerebrovascular events (mortality, non-fatal MI; CVA); *Rate*, proportion in group with condition present

### Graft patency

At 3 months, 316 patients survived and all were asymptomatic. Among these patients, 239 patients (75.6%) (120 patients in off-pump group and 119 patients in on-pump group) turned up for graft evaluation. Of the 239 patients (849 grafts), 190 patients (684 grafts) underwent graft evaluation by MDCT [off-pump; 96 patients (339 grafts) and on-pump; 94 patients (345 grafts)] and 49 patients (165 grafts) [(off-pump; 24patients (81 grafts) and on-pump; 25 patients (84 grafts)] underwent catheter CAG. The modality of graft evaluation was not uniform due to non-availability of MDCT facility at some centers where the graft evaluation was done by catheter CAG. The baseline characteristics were comparable between patients who underwent graft evaluation and who declined graft evaluation. However, the patients who underwent graft evaluation are younger than the patients who declined graft evaluation (57.93 ± 7.18 vs 59.85 ± 6.89, *p* = 0.03) (Table 5).

**Table 5:** Comparison of baselines variables between the patients in whom angio is performed and not performed

		Angio not done (81) *n* (%)	Angio done (239) *n* (%)	*p* value
Age (mean ± SD)		59.85 ± 6.89	57.93 ± 7.18	0.037
Sex	Female	12 (14.81)	24 (10.04)	0.24
	Male	69 (85.19)	215 (89.96)	
Smoking	No	69 (85.19)	208 (87.03)	0.67
	Yes	12 (14.81)	31 (12.97)	
Hypertension	No	26 (32.10)	84 (35.15)	0.61
	Yes	55 (67.90)	155 (64.85)	
Diabetes mellitus	No	34 (41.98)	99 (41.42)	0.93
	Yes	47 (58.02)	140 (58.58)	
Dyslipidemia	No	40 (49.38)	9 (40.59)	0.16
	Yes	41 (50.62)	142 (59.41)	
COPD	No	80 (98.77)	234 (97.91)	> 0.99*
	Yes	1 (1.23)	5 (2.09)	
Carotid artery stenosis > 60%	No	80 (98.77)	238 (99.58)	0.44*
	Yes	1 (1.23)	1 (0.42)	
Transient ischemic attack	No	81 (100.00)	237 (99.16)	> 0.99*
	Yes	0 (0.00)	2 (0.84)	
Cerebro vascular accident	No	73 (97.53)	235 (98.33)	0.64*
	Yes	2 (2.47)	4 (1.67)	
Myocardial infarction	No	59 (72.84)	161 (67.36)	0.35
	Yes	22 (27.16)	78 (32.64)	
Prior PTCA	No	78 (96.30)	227 (94.98)	0.76*
	Yes	3 (3.70)	12 (5.02)	
Left main disease	No	68 (83.95)	202 (84.52)	0.90
	Yes	13 (16.05)	37 (15.48)	
Surgery	Off-pump	38 (46.91)	120 (50.21)	0.60
	On-pump	43 (53.09)	119 (49.79)	

*Fisher exact test used

*COPD*, chronic obstructive pulmonary disorder; *PTCA*, percutaneous transluminal coronary angioplasty

**Fig. 2:**
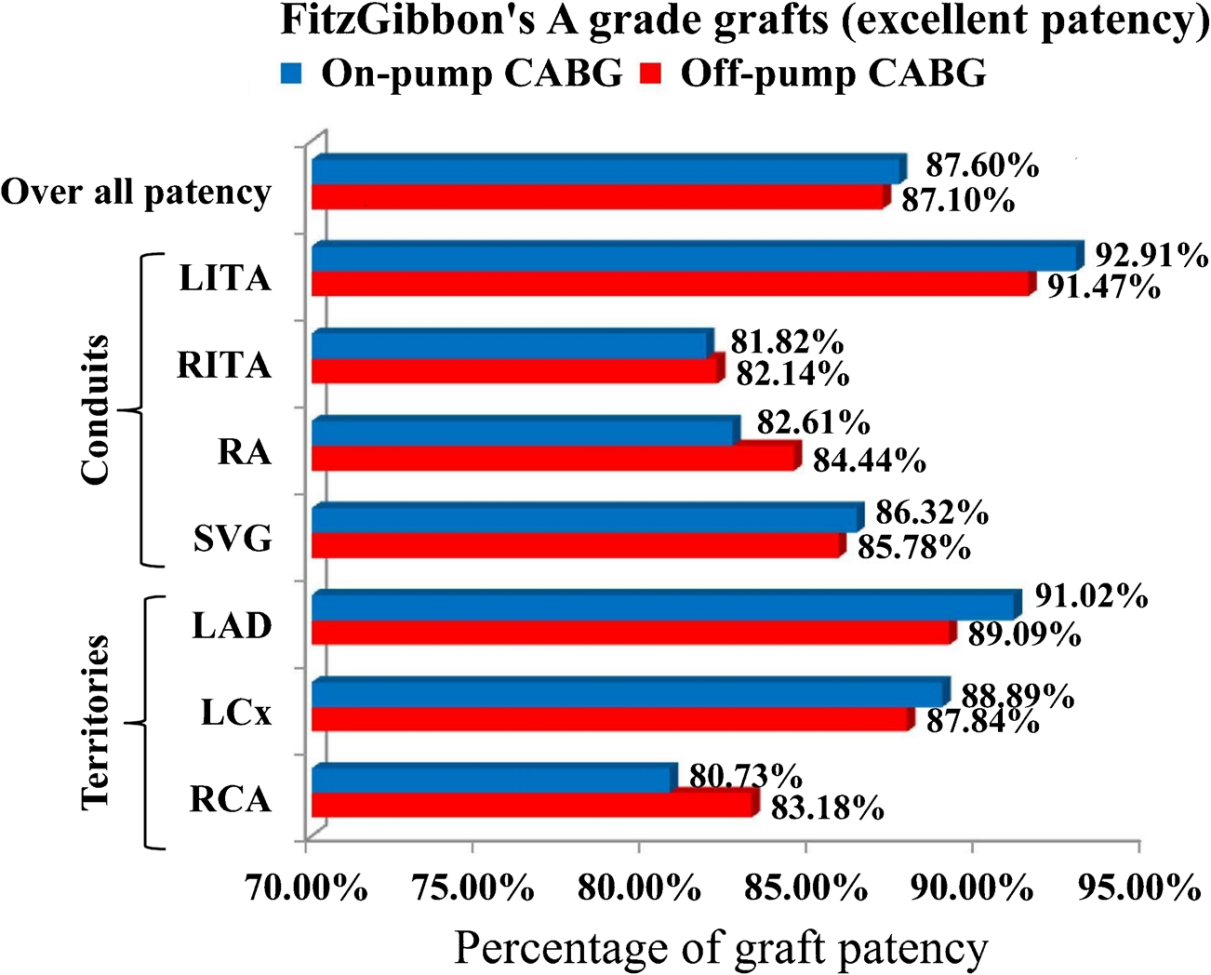
Graft patency rates between off-pump and on-pump CABG

**Fig. 3:**
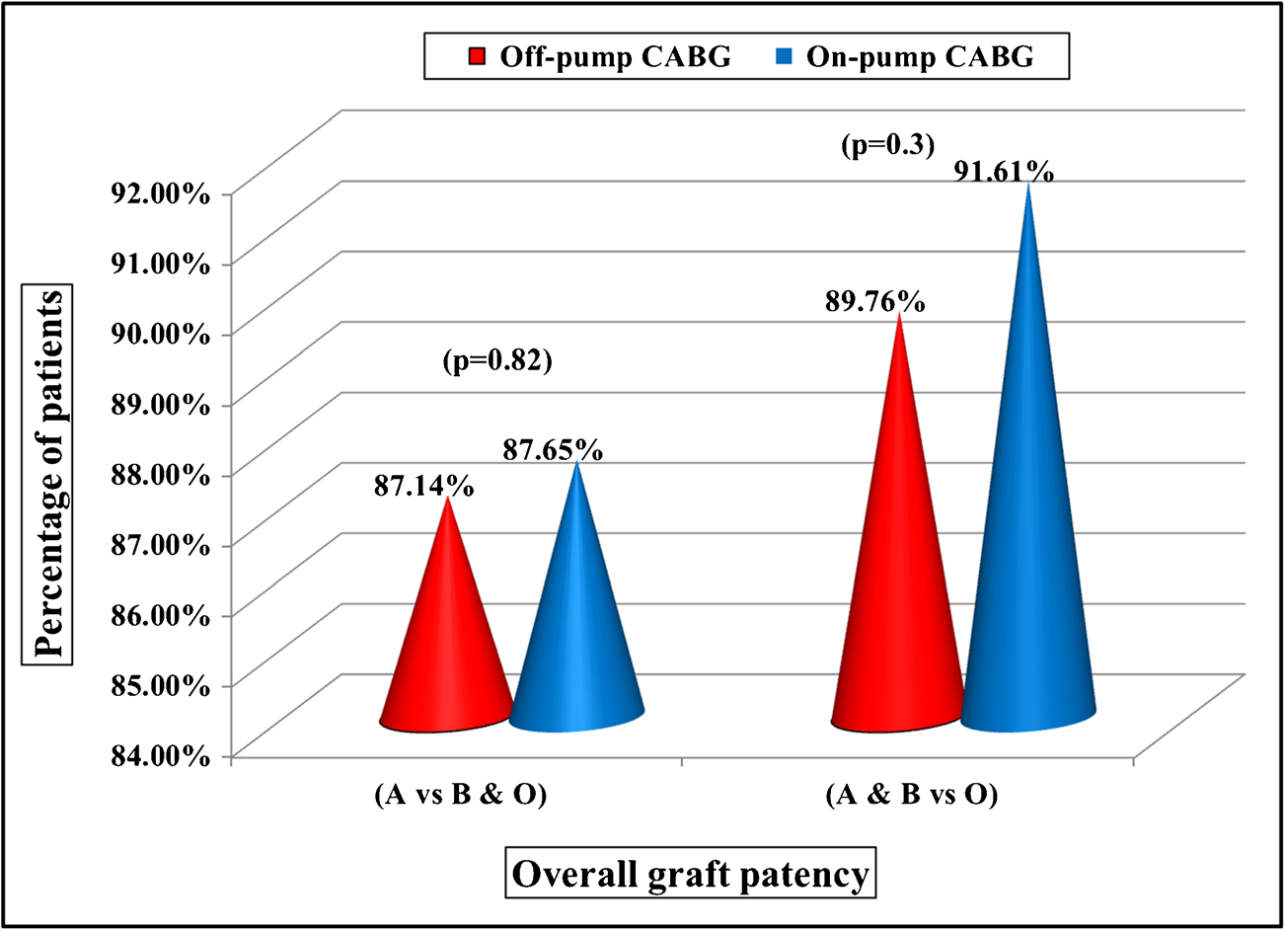
Overall graft patency showing patency rates of A vs B and O and A and B vs O

The graft evaluation was made similar to Fitzgibbon’s grading (A, B, and O grade grafts). The analysis was made by comparing A grade (excellent patency) grafts vs B and O grade (stenosed and occluded) grafts. In off-pump group (120 patients) out of 420 grafts, 366 (87.1%) grafts were grade A (excellent patency) grafts and in on-pump group (119 patients) out of 420 grafts, 376 (87.6%) grafts were grade A (excellent patency) grafts (*p* = 0.82). There was no significant deference in overall patency rates between the two techniques (Fig. 2). The analysis was also made comparing A and B vs O grade grafts. In on-pump group out of 429 grafts, 393 (91.6%) were grade A and B grafts and in off-pump group out of 420 grafts, 377 (89.76%) grafts were grade A and B grafts (*p* = 0.3) (Fig. 3).

The comparison between A grade grafts vs B and O grade grafts showed that there was no significant difference in the patency rates of various bypass conduits [LITA: off-pump 118/129 (91.4%) vs on-pump 118/127 (92.9%), (*p* = 0.66); RITA: off-pump 23/28 (82.1%) vs on-pump 18/22 (81.8%), (*p* = 0.97); radial artery (RA) graft: off-pump 38/45 (84.4%) vs on-pump 38/46 (82.6%), (*p* = 0.81); saphenous vein graft (SVG): off-pump 187/218 (85.8%) vs on-pump 202/234 (86.3%, (*p* = 0.86)] between the groups. Among the 3 coronary artery territories, patency rates of grafts were also similar [left anterior descending artery (LAD) territory: off-pump 147/165 (89.0%) vs on-pump 152/167 (91.0%), (*p* = 0.55); left circumflex artery (LCx) territory: off-pump 130/148 (87.4%) vs on-pump 136/153 (88.9%), (*p* = 0.77), and right coronary artery (RCA) territory: off-pump 89/107 (83.1%) vs on-pump 88/108 (80.7%), (*p* = 0.64)] (Table 6) and (Fig. 2).

**Table 6:** Rates of grade A grafts (excellent patency similar to Fitzgibbon’s grading) in various conduits and territories

Conduit	Grade A grafts/total grafts (percentage)	Off-pump CABG Grade A grafts/total grafts (percentage)	On-pump CABG Grade A grafts/total grafts (percentage)	*p* value
LITA	236/256 (92.10%)	118/129 (91.47%)	118/127 (92.91%)	0.66
RITA	41/50 (82.0%)	23/28 (82.14%)	18/22 (81.82%)	0.97
RA	76/91 (83.50%)	38/45 (84.44%)	38/46 (82.61%)	0.81
SVG	389/452 (86.06%)	187/218 (85.78%)	202/234 (86.32%)	0.86
All conduits	742/849 (87.39%)	366/420 (87.14%)	376/429 (87.65%)	0.82
Territories	Grade A grafts/total grafts (percentage)	Off-pump CABG Grade A grafts/total grafts (percentage)	On-pump CABG Grade A grafts/total grafts (percentage)	*p* value
LAD	299/332 (90.06%)	147/165 (89.09%)	152/167 (91.02%)	0.55
LCX	266/301 (88.37%)	130/148 (87.84%)	136/153 (88.89%)	0.77
RCA	177/216 (81.94%)	89/107 (83.18%)	88/108 (80.73%)	0.64
All territories	742/849 (87.39%)	366/420 (87.14%)	376/429 (87.65%)	0.82

*CABG*, coronary artery bypass grafting; *LAD*, left anterior descending artery; *LCx*, left circumflex artery; *LITA*, left internal thoracic artery; *RA*, radial artery; *RCA*, right coronary artery; *RITA*, right internal thoracic artery; *SVG*, saphenous vein graft

When comparison was made between A and B grade grafts vs O grade grafts, there was no significant difference in the patency rates of various bypass conduits. [LITA: off-pump 122/129 (94.5%) vs on-pump 122/127 (96.06%), (*p* = 0.57); RITA: off-pump 23/28 (82.1%) vs on-pump 18/22 (81.8%), (*p* = 0.97); RA graft: off-pump 39/45 (86.67%) vs on-pump 41/46 (89.13%), (*p* = 0.71); SVG: off-pump 193/218 (88.53%) vs on-pump 212/234 (90.6%), (*p* = 0.47)] between the groups. Among the 3 coronary artery territories, patency rates of grafts were also similar [LAD territory: off-pump 152/165 (92.12%) vs on-pump 157/167 (94.01%), (*p* = 0.49); LCx territory: off-pump 133/148 (89.86%) vs on-pump 141/153 (92.15%), (*p* = 0.48), and RCA territory: off-pump 92/107 (85.98%) vs on-pump 95/109 (87.15%), (*p* = 0.8)] between the groups .

### Analysis for non-inferiority in terms of graft patency in off-pump CABG

Of 119 patients in on-pump CABG group who underwent graft evaluation, 111 (93%) patients had grade A grafts and of 120 patients in off-pump CABG group, and 113 (94%) patients had grade A grafts. Of 429 grafts in on-pump CABG group, 376 (87%) grafts were grade A grafts and of 420 grafts in off-pump CABG group 366 (87%) grafts were grade A grafts (Table 7). The results show that off-pump CABG is non-inferior to on-pump CABG with a significant *p* value (*p* = 0.0001), thus rejecting the null hypothesis (off-pump CABG is inferior to on-pump CABG).

**Table 7:** Non-inferiority test results for all grafts and subjects

Variable	Off-pump group		On-pump group		95% CI for difference		NI margin (% of on-pump group)		*p* value	Non-inferiority attained (yes/no)
	Total	Patent *n* (%)	Total	Patent *n* (%)	Low	Upper				
Grafts	420	366 (87)	429	376 (87)	− 0.050	0.040	10	− 0.0876	0.0001	Yes
							5	− 0.0438	0.0444	Yes
Subjects	120	113 (94)	119	111 (93)	− 0.053	0.070	10	− 0.0932	< 0.0006	Yes
							5	− 0.0466	< 0.0385	Yes

*CI*, confidence interval, *n*, sample of population; *NI*, non-inferiority

## Discussion

Graft occlusion is one of the major determinants of clinical prognosis after CABG and is measured by re-intervention rates and survival. In countries like India, Japan, and Brazil with an off-pump CABG adoption rate in excess of 50% [2, 7], it is prudent to query the non-inferiority of this technique as compared with on-pump CABG. Angiographic evaluation was performed at 3 months considering that edema at the site of anastomosis would resolve by that time and it would be too early to have graft attrition. In the present study, the graft patency rates were similar between off-pump and on-pump technique as in the Surgical Management of Arterial Revascularization Therapies (SMART) trial [6] where angiography was done at 30 days and at 1 year. But, our study differs from the angiographic patency rates of Khan et al. [3] and ROOBY trial [1] which reported lower patency rates with off-pump technique. The possible reason for lower graft patency in off-pump technique in ROOBY trial [1] was thought to be the inexperience of the surgeons involved in the study as evidenced by a higher conversion rates from off-pump to on-pump technique (12.7%). In our study, the conversion rate was 1.27%. There has been no significant difference in the index of completeness of revascularization between the groups in our study though higher rates of incomplete revascularization were reported by several authors [10, 11]. This can also be attributed to the experience of the surgeons who participated in the study. The graft patency rates at 3 months with various bypass conduits among the different coronary territories were similar between off and on-pump groups, which coincide with the results of Puskas and colleagues [6] and Magee et al. [12] who reported similar patency rates at 1 year.

Our study is concurrent with Diegeler et al. [13] in GOPCABEtrial, Lamy et al. [14] in CORONARY trial, and Taggart et al. [15] in Arterial Revascularization Trial (ART) which reported no significant difference in the rate of composite outcome of death at 30 days. In a recent report, Chikwe et al. [16] reported that off-pump CABG was associated with an increased incidence of incomplete revascularization, need for repeated revascularization, and mortality in early outcomes and at 10 years compared with on-pump CABG. However, they have not assessed the graft patency in their patients. But, quite a few grafts that fail, do so with little immediate clinical consequence to the patient. Our study demonstrated marginally lower overall graft patency rates (excellent grafts 87%), which may be attributable to complex coronary anatomy with higher Syntax score in our patient population, and smaller coronary artery size. Some patients with target artery stenosis of 70 to 90% sub critical stenosis have received RITA graft to posterior descending artery (PDA)/distal RCA as the most distant distal anastomoses. This could have probably contributed to the lower graft patency of RITA grafts. Similar patency rates of sequentially anastomosed RITA graft to PDA were reported by Glineur et al. [17]. Further, this study analyzed grade A grafts as patent grafts and B and O grade grafts as occluded grafts in contrast to other studies which compared A and B grade grafts vs O grafts [1, 6, 18, 19]. The cohorts of patients are being followed up yearly for clinical evaluation and angiographic graft evaluation is contemplated at 5 years.

### Limitations

The evaluation of graft patency was not uniform and was done by either MDCT or catheter CAG. In 80% of patients, graft evaluation was done by MDCT, which despite its accuracy for detection of bypass graft occlusions does not provide information on graft flow but was used in recent large studies comparing late off-pump and on-pump graft patency trials [20, 21]. Less than 10% of screened patients were enrolled in the study due to difficulty in obtaining the consent for early angiographic evaluation. Only 75% of patients (849 grafts) were subjected to angiographic graft evaluation. Only low-risk patients were enrolled in this study.

## Conclusions

The study demonstrates that there is no significant difference in overall graft patency rates at 3 months between off-pump and on-pump CABG groups when performed by experienced surgeons who have a higher adoption of this strategy. Further, this study shows no difference in the graft patency among the 3 coronary artery territories and the different types of conduits between off-pump and on-pump CABG patients. At 3 months follow-up, off-pump CABG was associated with a fewer MACCE compared to on-pump CABG.

## Electronic supplementary material

ESM 1 (PDF 53 kb)

ESM 2 (PDF 192 kb)
