# Supplementary material for: Graft patency at 3 months after off- and on-pump coronary bypass surgery: a randomized trial
Source: Indian J Thorac Cardiovasc Surg. 2019 Oct 28;36(2):93–104. doi: 10.1007/s12055-019-00869-0 (PMC7525555; doi:10.1007/s12055-019-00869-0)
Supplement: Supplementary file 1 — (PDF 53 kb) [file 12055_2019_869_MOESM1_ESM.pdf]

Clinical Trial Details (PDF Generation Date :- Sun, 30 Sep 2018 08:47:10 GMT)

**CTRI Number**	CTRI/2017/10/010030 [Registered on: 06/10/2017] - **Trial Registered Retrospectively**
**Last Modified On**	10/10/2017
**Post Graduate Thesis**	No
**Type of Trial**	Interventional
**Type of Study**	Surgical/Anesthesia
**Study Design**	Randomized, Crossover Trial
**Public Title of Study**	Comparison of graft patency in on-pump heart surgery versus off-pump heart Surgery
**Scientific Title of Study**	Prospective Randomized comparison of Off-pump and On-pump Multi-vessel Coronary artery bypass surgery To Evaluate outcomes and graft patency

Secondary IDs if Any

Secondary ID	Identifier
NIL	NIL

Details of Principal Investigator or overall Trial Coordinator (multi-center study)

Details of Principal Investigator	
**Name**	Dr Lokeswara Rao Sajja
**Designation**	Sr. Consultant Cardiothoracic Surgeon
**Affiliation**	STAR Hospitals
**Address**	8-2-596/5, Road No.10, Banjara Hills, Hyderabad<br>Hyderabad<br>ANDHRA PRADESH<br>500034<br>India
**Phone**	91-9849028317
**Fax**	
**Email**	sajjalr@yahoo.com

Details Contact Person (Scientific Query)

Details Contact Person (Scientific Query)	
**Name**	Dr Lokeswara Rao Sajja
**Designation**	Sr. Consultant Cardiothoracic Surgeon
**Affiliation**	STAR Hospitals
**Address**	8-2-596/5, Road No.10, Banjara Hills, Hyderabad<br><br>ANDHRA PRADESH<br>500034<br>India
**Phone**	91-9849028317
**Fax**	
**Email**	sajjalr@yahoo.com

Details Contact Person (Public Query)

Details Contact Person (Public Query)	
**Name**	Dr Lokeswara Rao Sajja
**Designation**	Sr. Consultant Cardiothoracic Surgeon
**Affiliation**	STAR Hospitals
**Address**	8-2-596/5, Road No.10, Banjara Hills, Hyderabad<br><br>ANDHRA PRADESH<br>500034<br>India
**Phone**	91-9849028317
**Fax**	
**Email**	sajjalr@yahoo.com

Source of Monetary or Material Support

Source of Monetary or Material Support
> None

Primary Sponsor

Primary Sponsor Details	
**Name**	Dr Lokeswara Rao Sajja
**Address**	Star Hospitals, 8-2-596/5, Road No.10, Banjara Hills, Hyderabad - 500034
**Type of Sponsor**	Other [Individual]

Details of Secondary Sponsor

Name	Address
Dr Gopichand Mannam	Star Hospitals, 8-2-596/5, Road No.10, Banjara Hills, Hyderabad - 500034
Dr Kunal Sarkar	Medica Superspeciality Hospital, 127-Mukundapur, EM bypass, Kolkata - 700025

Countries of Recruitment

List of Countries
India

Sites of Study

Name of Principal Investigator	Name of Site	Site Address	Phone/Fax/Email
Dr Sanjeeth Peter	DDMM Heart Institute	Mission Road, Nadiad - 387002,<br>Ahmadabad<br>GUJARAT	91-9825929270<br><br>sanjeethpeter@gmail.com
Dr Anvay Mulay	Fortis Multispeciality Hospital	Mulund West, Goregaon Link Road, Mumbai - 400078<br>Mumbai (Suburban)<br>MAHARASHTRA	91-9320174093<br><br>anvay03@gmail.com
Dr Chandra Sekhar Padmanabhan	GKNM Hospital	PB No:6327, Nethaji Road, Pappanaickenpalayam, Coimbatore - 641 037<br>Coimbatore<br>TAMIL NADU	91-9443047152<br><br>chanpad@gmail.com
Dr KV Krishna Kumar	Krishna Intitute of Medical Sciences (KIMS)	1-8-31/1, Minister Road, Krishna Nagar Colony, Begumpet, Secunderabad<br>Hyderabad<br>ANDHRA PRADESH	91-9849012912<br><br>vkkumark@hotmail.com
Dr Kunal Sarkar	Medica Super Speciality Hospital	127 Mukundapur, EM Bypass, Kolkata - 700025<br>Kolkata<br>WEST BENGAL	91-9830080006<br><br>kunal.cardiac@gmail.com
Dr Gopichand Mannam	STAR Hospitals	8-2-596/5, Road No.10, Banjara Hills, Hyderabad - 500034<br>Hyderabad<br>ANDHRA PRADESH	91-9849027027<br>91-40-23356788<br>gopi.mannam@gmail.com
Dr Lokeswara Rao Sajja	STAR Hospitals	8-2-596/5, Road No.10, Banjara Hills, Hyderabad - 500034<br>Hyderabad<br>ANDHRA PRADESH	91-9849028317<br>91-40-23356788<br>sajjalr@yahoo.com

Details of Ethics Committee

Name of Committee	Approval Status	Date of Approval	Is Independent Ethics Committee?

Institutional Ethics Committee - DDMM	Approved	05/01/2016	No
Institutional Ethics Committee - Fortis Mumbai	Approved	31/03/2016	No
Institutional Ethics Committee - GKNM	Approved	18/12/2015	No
Institutional Ethics Committee - Medica	Submittted/Under Review	No Date Specified	No
Institutional Ethics Committee - STAR	Approved	21/11/2015	No
Institutional Ethics Committee - STAR	Approved	21/11/2015	No
KIMS Foundation & Research Center	Approved	04/02/2016	No

Regulatory Clearance Status from DCGI

Status	Date
Not Applicable	No Date Specified

Health Condition / Problems Studied

Health Type	Condition
Patients	Multi-vessel Coronary Artery Disease

Intervention / Comparator Agent

Type	Name	Details
Intervention	Off-Pump CABG	Beating Heart Surgery
Comparator Agent	On-Pump CABG	Heart Surgery on Cardiopulmonary Bypass

Inclusion Criteria

Inclusion Criteria	
**Age From**	21.00 Year(s)
**Age To**	70.00 Year(s)
**Gender**	Both
**Details**	1. Male or female aged ?21years to ?70years<br><br>2. Able to provide written informed consent<br><br>3. Patients with significant CAD with significant triple vessel disease or LMCA stenosis angiographically documented ischemia due to multi vessel coronary artery disease<br><br>4. Require isolated CABG<br>5. LVEF ? 40 %

Exclusion Criteria

Exclusion Criteria	
**Details**	1. CABG with concomitant valvular procedures<br><br>2. CABG with concomitant repair of congenital heart disease.<br><br>3. Contra-indications to off-pump CABG or on-pump CABG (calcified aorta, intramuscular LAD, calcified coronaries, small target vessels, resection of ventricular aneurysm)<br><br>4. Severe congestive heart failure, Ney York Heart Association (NYHA) Class III or IV or pulmonary edema.<br><br>5. Chronic atrial fibrillation<br><br>6. Allergy to contrast material

		7. Prior valve replacement or Redo CABG<br><br>8. Prior percutaneous coronary intervention (PCI) with stent implantation within 3 months<br><br>9. Concomitant medical disorders making clinical follow-up at least 3months unlikely or impossible e.g. neoplastic, hepatic, or other severe disease<br><br>10. Emergency CABG<br><br>11. LVEF ?39 %<br><br>12. Unable to give consent

**Method of Generating Random Sequence**	Stratified block randomization
**Method of Concealment**	Sequentially numbered, sealed, opaque envelopes
**Blinding/Masking**	Open Label

Primary Outcome

Outcome	Timepoints
The primary outcome is the assessment of Conventional coronary angiographic patency of the grafts at 3 months.	The primary outcome is the assessment of Conventional coronary angiographic patency of the grafts at 3 months.

Secondary Outcome

Outcome	Timepoints
The secondary outcome is the occurrence of the composite of cardiovascular death, stroke, nonfatal MI, or new onset renal failure (AKI) requiring CRRT.	At 3 months

**Target Sample Size**	**Total Sample Size=**400<br>**Sample Size from India=**400
**Phase of Trial**	N/A
**Date of First Enrollment (India)**	23/03/2016
**Date of First Enrollment (Global)**	No Date Specified
**Estimated Duration of Trial**	**Years=**1<br>**Months=**0<br>**Days=**0
**Recruitment Status of Trial (Global)**	Not Applicable
**Recruitment Status of Trial (India)**	Completed
**Publication Details**	none yet
**Brief Summary**	This is a prospective randomized multicenter trial comparing off-pump CABG and on-pump CABG in 400 indian patients (200 patients in each group) undergoing isolated primary CABG surgery will be enrolled in the study from 6 centers in India.<br><br>The study will compare graft patency of off-pump and on-pump coronary artery bypass surgery procedures in patients with multi-vessel coronary artery disease who referred for isolated CABG in Indian population due to inconsistency in the literature as to the relative bene?ts of on-pump and

	off-pump CABG. The purpose is to provide efficacy and graft patency data in Indian population. The primary objective of this study is to assess coronary artery bypass graft patency evaluation at 3 months by Conventional coronary angiographic patency in patients randomized to off-pump and on-pump multi vessel coronary artery bypass grafting (CABG). The secondary objective is to evaluate outcomes of CABG at 30 days, MACCE up to 3 months, to assess functional status and Quality of life at 3 months.
