# Supplementary material for: Graft patency at 3 months after off- and on-pump coronary bypass surgery: a randomized trial
Source: Indian J Thorac Cardiovasc Surg. 2019 Oct 28;36(2):93–104. doi: 10.1007/s12055-019-00869-0 (PMC7525555; doi:10.1007/s12055-019-00869-0)
Supplement: Supplementary file 2 — (PDF 192 kb) [file 12055_2019_869_MOESM2_ESM.pdf]

International Clinical Trials Registry Platform
Search Portal

Main

Note: This record shows only 21 elements of the WHO Trial Registration Data Set. To view changes that have been made to the source record, or for additional information about this trial, click on the URL below to go to the source record in the primary register.

**Register:**	CTRI
**Last refreshed on:**	11 September 2018
**Main ID:**	CTRI/2017/10/010030
**Date of registration:**	06-10-2017
**Prospective Registration:**	No
**Primary sponsor:**	Dr Lokeswara Rao Sajja
**Public title:**	Comparison of graft patency in on-pump heart surgery versus off-pump heart Surgery
**Scientific title:**	Prospective Randomized comparison of Off-pump and On-pump Multi-vessel Coronary artery bypass surgery To Evaluate outcomes and graft patency
**Date of first enrolment:**	23-03-2016
**Target sample size:**	400
**Recruitment status:**	Completed
**URL:**	http://www.ctri.nic.in/Clinicaltrials/pmaindet2.php?trialid=14112
**Study type:**	Interventional
**Study design:**	Randomized, Crossover Trial Method of generating randomization sequence:Stratified block randomization Method of allocation concealment:Sequentially numbered, sealed, opaque envelopes Blinding and masking:Open Label
**Phase:**	N/A

Countries of recruitment

India

Contacts

**Name:**	Dr Lokeswara Rao Sajja	**Name:**	Dr Lokeswara Rao Sajja
**Address:**	8-2-596/5, Road No.10, Banjara Hills, Hyderabad 500034 Bangalore, ANDHRA PRADESH India	**Address:**	8-2-596/5, Road No.10, Banjara Hills, Hyderabad 500034 Bangalore, ANDHRA PRADESH India
**Telephone:**	91-9849028317	**Telephone:**	91-9849028317
**Email:**	sajjalr@yahoo.com	**Email:**	sajjalr@yahoo.com
**Affiliation:**	STAR Hospitals	**Affiliation:**	STAR Hospitals

Key inclusion & exclusion criteria

Inclusion criteria: 1. Male or female aged â?¥21years to â?¤70years

2. Able to provide written informed consent

3. Patients with significant CAD with significant triple vessel disease or LMCA stenosis angiographically documented ischemia due to multi vessel coronary artery disease

4. Require isolated CABG

5. LVEF â?¥ 40 %

Exclusion criteria: 1. CABG with concomitant valvular procedures

2. CABG with concomitant repair of congenital heart disease.

3. Contra-indications to off-pump CABG or on-pump CABG (calcified aorta, intramuscular LAD, calcified coronaries, small target vessels, resection of ventricular aneurysm)

4. Severe congestive heart failure, Ney York Heart Association (NYHA) Class III or IV or pulmonary edema.

5. Chronic atrial fibrillation

6. Allergy to contrast material

7. Prior valve replacement or Redo CABG

8. Prior percutaneous coronary intervention (PCI) with stent implantation within 3 months

9. Concomitant medical disorders making clinical follow-up at least 3months unlikely or impossible e.g. neoplastic, hepatic, or other severe disease

10. Emergency CABG

11. LVEF â?¤39 %

12. Unable to give consent

Age minimum:
Age maximum:
Gender:

Health Condition(s) or Problem(s) studied

Multi-vessel Coronary Artery Disease

Intervention(s)

Intervention1: Off-Pump CABG: Beating Heart Surgery
Control Intervention1: On-Pump CABG: Heart Surgery on Cardiopulmonary Bypass

Primary Outcome(s)

The primary outcome is the assessment of Conventional coronary angiographic patency of the grafts at 3 months.------Timepoint: The primary outcome is the assessment of Conventional coronary angiographic patency of the grafts at 3 months.

Secondary Outcome(s)

The secondary outcome is the occurrence of the composite of cardiovascular death, stroke, nonfatal MI, or new onset renal failure (AKI) requiring CRRT.------Timepoint: At 3 months

Secondary ID(s)

NIL

Source(s) of Monetary Support

None

Secondary Sponsor(s)

Dr Gopichand Mannam

Dr Kunal Sarkar

Results

Results available:
Date Posted:
URL:

Disclaimer: Trials posted on this search portal are not endorsed by WHO, but are provided as a service to our users. In no event shall the World Health Organization be liable for any damages arising from the use of the information linked to in this section. None of the information obtained through use of the search portal should in any way be used in clinical care without consulting a physician or licensed health professional. WHO is not responsible for the accuracy, completeness and/or use made of the content displayed for any trial record.

Copyright - World Health Organization - Version 3.6 - Version history
